# Global Research Priorities to Better Understand the Burden of Iatrogenic Harm in Primary Care: An International Delphi Exercise

**DOI:** 10.1371/journal.pmed.1001554

**Published:** 2013-11-19

**Authors:** Kathrin M. Cresswell, Sukhmeet S. Panesar, Sarah A. Salvilla, Andrew Carson-Stevens, Itziar Larizgoitia, Liam J. Donaldson, David Bates, Aziz Sheikh, on behalf of the World Health Organization's (WHO) Safer Primary Care Expert Working Group

**Affiliations:** 1 The School of Health in Social Science, The University of Edinburgh, Edinburgh, United Kingdom; 2 Centre for Population Health Sciences, The University of Edinburgh, Edinburgh, United Kingdom; 3 Cochrane Institute of Primary Care & Public Health, School of Medicine, Cardiff University, Cardiff, United Kingdom; 4 WHO, Geneva, Switzerland; 5 Department of Surgery and Cancer, Imperial College London, London, United Kingdom; 6 Division of General Internal Medicine, Brigham and Women's Hospital, Boston, Massachusetts, United States of America; 7 Harvard Medical School, Boston, Massachusetts, United States of America; 8 Department of Health Policy and Management, Boston, Massachusetts, United States of America

## Abstract

Using a modified Delphi exercise, Aziz Sheikh and colleagues identify research priorities for patient safety research in primary care contexts.

*Please see later in the article for the Editors' Summary*

**Summary Points**

- There is a need to identify and reach agreement on key foci for patient safety research in primary care contexts and understand how these priorities differ between low-, middle-, and high-income settings.
- We conducted a modified Delphi exercise, which was distributed to an international panel of experts in patient safety and primary care.
- Family practice and pharmacy were considered the main contexts on which to focus attention in order to advance patient safety in primary care across all income categories. Other clinical contexts prioritised included community midwifery and nursing in low-income countries and care homes in high-income countries.
- The sources of patient safety incidents requiring further study across all economic settings that were identified were communication between health care professionals and with patients, teamwork within the health care team, laboratory and diagnostic imaging investigations, issues relating to data management, transitions between different care settings, and chart/patient record completeness.
- This work lays the foundation for a range of research initiatives that aim to promote a more comprehensive appreciation of the burden of unsafe primary care, develop understanding of the main areas of risk, and identify interventions that can enhance the safety of primary care provision internationally.

## Tackling the Provision of Unsafe Primary Care Internationally

It is now well established that medical errors are common and that these can result in considerable morbidity and mortality [1]–[3]. Much of this evidence, however, comes from hospital settings in industrialised countries where considerable progress has been made in describing the epidemiology of errors, understanding underlying contributing factors, and, more recently, taking steps to intervene to enhance patient safety [4].

In contrast, much less is known about the frequency of patient safety incidents and preventability of harm in primary care, particularly in low- and middle-income countries (Box 1). This is of concern as, in many parts of the world, primary care-based services now provide the first point of contact with health systems and often play a key role in coordinating more specialist care provision [5]–[8]. The increasing move to primary care-based health systems internationally [9],[10] adds further impetus to the urgent need for research into the frequency and preventability of patient safety incidents, but this is complicated by the considerable variation in population needs, economic and political circumstances, structures of health systems, and manifestations of primary care globally.

**Box 1. Glossary of Key Definitions**

**Primary care**

“Primary care is the provision of integrated, accessible health care services by clinicians who are accountable for:

addressing a large majority of personal health care needs

developing a sustained partnership with patients

practicing in the context of family and community” [10].

**Patient safety**

“Patient safety is the reduction of risk of unnecessary harm associated with health care to an acceptable minimum. An acceptable minimum refers to the collective notions of given current knowledge, resources available and the context in which care was delivered weighed against the risk of non-treatment or other treatment” [28].

**Harm**

“Harm implies impairment of structure or function of the body and/or any deleterious effect arising there from, including disease, injury, suffering, disability and death, and may be physical, social or psychological. Disease is a physiological or psychological dysfunction. Injury is damage to tissues caused by an agent or event and suffering is the experience of anything subjectively unpleasant. Suffering includes pain, malaise, nausea, depression, agitation, alarm, fear and grief. Disability implies any type of impairment of body structure or function, activity limitation and/or restriction of participation in society, associated with past or present harm” [28].

**Low-, middle-, and high-income countries**

“For operational and analytical purposes, the World Bank's main criterion for classifying economies is gross national income (GNI) per capita. In previous editions of our publications, this term was referred to as gross national product (GNP). Based on its GNI per capita, every economy is classified as low income, middle income (subdivided into lower middle and upper middle), or high income” [29].

In an attempt to support the development of a more comprehensive evidence-base, the World Health Organization (WHO) convened an international group of experts to discuss, debate, and advise on directions to bridge knowledge gaps around safe primary care, which would also serve to catalyse research in these areas internationally. A key strand of this foundational work was to identify a shared vision on relevant contexts of primary care and areas that would need further study to better understand the burden of harm in primary care settings internationally.

## Developing Agreement on Primary Care Contexts and Priority Areas

We conducted a three-stage modified Delphi exercise, aiming to seek agreement on the most important contexts of primary care and the potential causes of patient safety incidents in different economic settings [11],[12]. This exercise was undertaken during a two-day expert meeting in February 2012 at the WHO headquarters in Geneva, Switzerland. AS, DB, and IL jointly chaired this meeting, which consisted of presentations, discussion groups, and plenary sessions focusing on understanding the challenges of assessing patient safety in primary care in low-, middle-, and high-income settings [13]–[15].

The Delphi technique has been widely used to help promote agreement amongst international experts. Key strengths of this process include the fact that it does not force consensus, but rather it can help to identify where agreement does and does not exist [16]–[19]. Although the general purpose and procedures have been retained in modified versions, some important differences in methods relate to: (1) approaches to managing interactions between experts, (2) the design of the initial item generation, and (3) feedback of individual scores [20]–[22].

## Identifying Experts

We identified experts from academic, policy, and clinical backgrounds with expertise relating to patient safety in primary care settings. This initial long-list was generated by drawing on existing WHO contacts, profiling academic institutions with relevant expertise in the area, identifying authors of published studies, and by searching Google Scholar. Selected experts were invited to participate at the meeting. Provisions were made to facilitate representation from low- and middle-income countries in different geographical regions, and by meeting travel costs where financial considerations were a possible barrier to attendance.

## Generating Candidate Statements and Prioritisation Exercise

Participants were provided with a review of the literature surrounding the frequency of patient safety incidents, burden of harm, and preventability of these incidents in primary care. Data were collected in three iterative stages with corresponding data collection forms (see Text S1). The forms were distributed and collected face-to-face by members of the research team (KMC, SAS, SSP, and ACS). Opportunity for free text comments was provided throughout and each participant was assigned a number for anonymisation purposes.

The forms included a list of candidate areas identified from the literature, which were grouped into three sections with corresponding statements for low-, middle-, and high-income countries (see Text S1). After piloting, the list was shared with the experts at the beginning of day 1 of the meeting, asking participants to add additional items. This list was amended on the basis of participant feedback.

The amended list formed the basis for the second round of data collection. Here, experts were asked to score items in terms of importance (frequency of occurrence, severity of outcome, preventability, inequity of occurrence) on a 9-point Likert-type scale for each income category ranging from 1 = “not important” to 9 = “extremely important” [15]. Data were independently scrutinised and transcribed by two members of the research team (KMC and ACS) into Microsoft Excel spread sheets and the median score for all items and the percentage agreement for items scoring 7, 8, or 9 (“usually important,” “very important,” and “extremely important,” respectively, i.e., the highest scores) were calculated.

The medians and percentage agreements obtained for each item were then included in the revised questionnaire that formed the basis for round 3 of data collection, giving participants the opportunity to revise their scoring on the basis of other participants' rankings. The third questionnaire was distributed and collected at the end of day 1, followed by calculation of the percentage agreement with individual items.

Items with an agreement of >80% in each section at the end of the Delphi exercise were fed back to participants on day 2 of the meeting. Rather than feeding back the actual distribution of panellists' ratings on the prior round, because of time and resource constraints the process fed back the median rating and percentage agreement of the prior round for the 7, 8, or 9 category as a proxy for the full distribution. This was followed by a plenary discussion, which gave participants the opportunity to collectively discuss emerging conclusions and recommendations. It enabled an exploration of areas of convergence and divergence across countries and perspectives, giving participants the opportunity to air concerns and discuss potential next steps.

## Overarching Considerations to Improve Understanding of the Extent of Unsafe Primary Care

**Figure 1:**
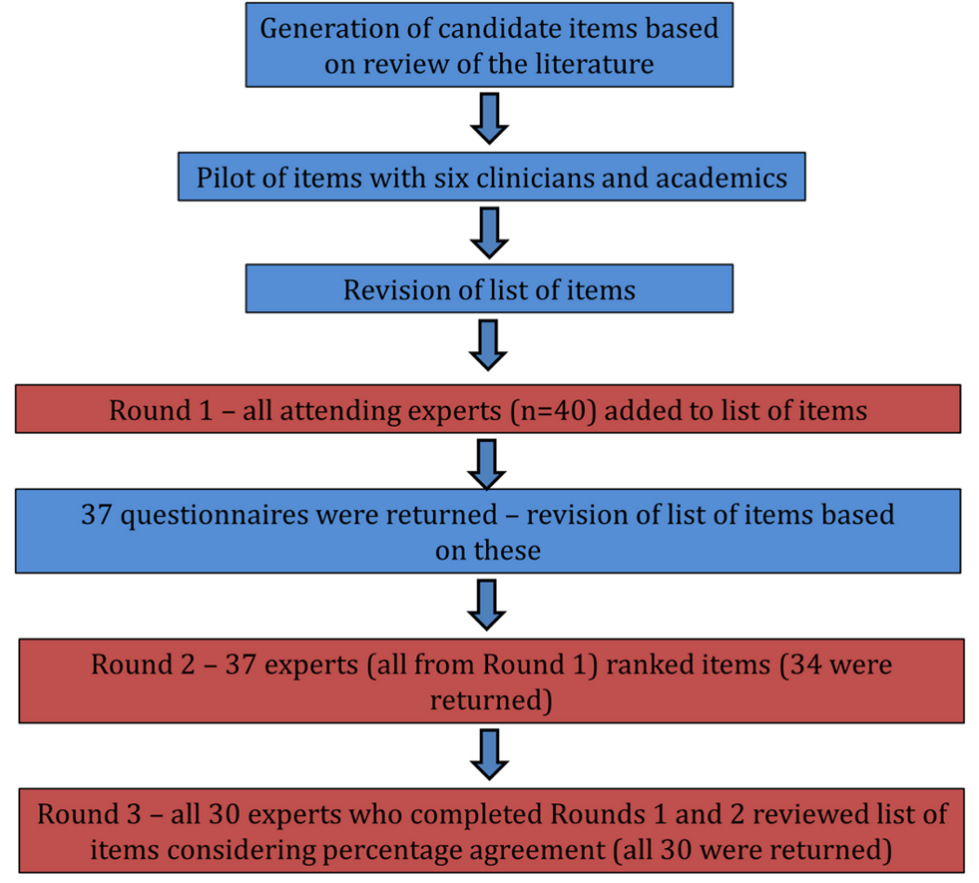
Flow-diagram of the three-stage modified Delphi process.

We distributed 40 questionnaires in round 1. Of these, 37 questionnaires were completed. Reasons for non-completion were participants either failing to return the questionnaire (*n* = 1) or leaving the meeting early (*n* = 2). No potential participant explicitly refused to participate in the study. Of the 37 questionnaires distributed in round 2, 34 were returned (non-responses were again mainly because some participants needed to leave the meeting early (*n* = 3)). In round 3, we distributed 30 questionnaires to the remaining participants (the other four had left the meeting), all of which were completed. The overall response rate was therefore 30/40 (75%; see Figure 1). Key characteristics of those who completed all three rounds of the Delphi exercise are detailed in Table 1.

**Table 1:** Key participant characteristics of experts in all three rounds of the modified Delphi exercise.

Round	Gender	Professional Background	Number of Countries Represented	Income Settings Represented
**1**	8 female29 male	8 academic (mostly doctors, some with pharmacy and nursing backgrounds)7 non-for-profit research10 health policy7 clinical (all doctors)5 academic/clinical	18	10 high-income5 middle-income3 low-income
**2**	8 female26 male	8 academic4 non-for-profit research10 health policy7 clinical5 academic/clinical	17	9 high-income5 middle-income3 low-income
**3**	7 female23 male	6 academic3 non-for-profit research10 health policy6 clinical5 academic/clinical	15	7 high-income5 middle-income3 low-income

Overall, there was over 80% agreement across 15 items in low-income country contexts, 16 items in middle-income country contexts, and 16 items in high-income country contexts. Family practice and pharmacy were important primary care contexts across all income categories (Table 2). Additional contexts identified as warranting particular attention were community midwifery and nursing in low-income countries, and care homes in high-income countries (Table 2).

**Table 2:** Primary care contexts that were considered to be important by over 80% of participants after round 3.

**Primary care contexts prioritised across income settings**Family practice^a^Pharmacy
**Primary care contexts prioritised in low-income settings**Community midwiferyCommunity nursing
**Primary care contexts prioritised in middle-income settings**Community nursing
**Primary care contexts prioritised in high-income settings**Care homes

a Family practice was assumed to include general practice, outpatient paediatrics, and outpatient internal medicine.

Tables 3 and 4 summarise the factors responsible for patient safety incidents that were identified as particularly needing further investigation across income settings. Important additional items in low- and middle-income settings included counterfeit drugs and errors in the execution of clinical tasks, whilst additional items in high-income settings were systems management and technology-related issues (Table 5).

**Table 3:** Causes of patient safety incidents and their associated harm that were considered in need of further study by over 80% of participants after round 3 across all income settings.

Chart/patient record completeness
Communication between health care professionals in the same team
Communication between health care professionals from different teams
Data management
Laboratory investigations
Teamwork
Transitions between different levels of care
Wrong or missed diagnoses
Wrong treatment decision

**Table 4:** Five key causes of patient safety incidents that required further study across countries with different levels of income.

Problems resulting from poor communication and teamwork
Ordering and interpretation of diagnostic imaging and laboratory investigations
Issues relating to data management
Managing transitions between different levels of care
Completeness of patient records

**Table 5:** Items relating to causes of patient safety incidents and associated harm that were considered to be important by over 80% of participants after round 3 in low-, middle-, and high-income settings.

**Causes of patient safety incidents in primary care prioritised in low-income settings**Counterfeit drugsExecution of a clinical task (errors when performing clinical tasks due to lack of knowledge and/or skills)
**Causes of patient safety incidents in primary care prioritised in middle-income settings**Communication between health care professionals and patientsCounterfeit drugsExecution of a clinical task (errors when performing clinical tasks due to lack of knowledge and/or skills)Higher-level systems management, e.g., human resourcesInformation technology and tools, e.g., checklists
**Causes of patient safety incidents in primary care prioritised in high-income settings**Communication between health care professionals and patientsDiagnostic imagingHigher-level systems management, e.g., human resourcesInformation technology and tools, e.g., checklists

Participants also prioritised the importance of cross-cutting systems' issues (Table 6). As can be seen, a range of interventional, regulatory, and methodological issues emerged; it is noteworthy that improved education and training for primary care workers received unanimous support.

**Table 6:** Cross-cutting items that were considered to be important to focus on by over 80% of participants after round 3.

Education and training
Data collection methods
Developing policy to promote patient safety
Raising the public profile of patient safety
Greater clarity on definitions of errors in primary care
Facilitating learning from errors
Regulations to ensure that systems to improve patient safety are put into practice
Improved typologies/taxonomies (better ways of classifying errors in primary care)

Overall, we identified family practice and pharmacy as the main contexts to focus attention on in order to advance patient safety in primary care across all income categories. The sources of patient safety incidents requiring further study identified across all economic settings were communication between health care professionals and with patients, teamwork within the health care team, laboratory and diagnostic imaging investigations, issues relating to data management, transitions between different care settings, and chart/patient record completeness.

## Strengths and Limitations of the Approach Employed

This work provides a foundation from which to focus efforts on how to better quantify the extent of iatrogenic harm in primary care and, in due course, to develop interventions to enhance the safety of primary care provision globally [5],[6]. The exercise allowed us to identify areas for research into safer primary care, focusing on areas with the greatest propensity for harm and where prevention was considered feasible. Incorporating this exercise within a two-day face-to-face meeting helped to ensure good response rates retaining the majority of participants, and allowed feeding back results to participants in real time, taking their comments into account in analysis activities and questionnaire design. The iterative element of the exercise helped to inform the meeting's proceedings and ensuing discussions. We provided participants with the opportunity to discuss emerging areas of agreement, thereby contributing to the face validity of the results. The different backgrounds and expertise of participants created the opportunity to explore the specific challenges associated with primary care provision across a range of geographical and political contexts.

Our work also has some important limitations. The total number of participants was limited by resource constraints. Furthermore, as the focus was on policy and research deliberations, we had an over-representation of high-income country-based scholars and doctors, potentially influencing the results in favour of medical concerns important to those working in industrialised countries. That said, it is important to note that community pharmacy, nursing, and midwifery emerged as consistent priority areas. Some participants acknowledged that they had limited insights into provision of care in low-income settings, which may have influenced their ability to make informed judgements. Expansion of this exercise, involving additional experts from wider professional domains and world settings may therefore generate additional important insights.

Because of resource and time constraints in collating data over a narrow time window, we were unable to include a reminder of participants' own prior ratings, which may to an extent have been mitigated by the fact that all three rounds were conducted on the same day. We were also unable to provide additional information on distributions of ratings that could point to potentially diverging ratings and hence disagreements amongst experts that might not be reflected in the summary scores. We did however examine the raw data from each round for ambiguous items, but did not detect any such instances.

Finally, it should be noted that some known important patient safety issues in primary care such as injection safety were not included in this exercise because of a lack of specific expertise among participants, though it was noted as an important gap [23].

## Implications and Unresolved Issues

Some of the existing knowledge underlying the measurement, causal factors, and interventions to enhance patient safety in primary care may be applicable across a wide array of income settings. This knowledge may be particularly relevant in relation to common contexts of care provision, including the central positioning of family practice and community pharmacy in health systems globally, although there may be other contextual and institutional factors at play that need to be better understood. The expert consultation strongly advocated the need for further research surrounding the frequency and preventability of patient safety incidents in primary care.

The discipline of patient safety is built on the premise that harm arises from errors in a multifactorial chain of events [24],[25]. The underlying assumption is that if systems (i.e., organisations and networks of organisations) and working conditions within these organisations can be optimised, then the occurrence of adverse events is less likely. This systems approach is increasingly being applied as the patient safety culture of institutions and systems matures. Our findings support this trend, as reflected in the cross-cutting areas identified that all relate to improving ways of working collaboratively [26],[27].

The work accomplished in this meeting can now be used as a starting point to inform and focus efforts in relation to epidemiological investigations that are urgently needed, particularly in low- and middle-income country contexts. These insights can then be used to develop interventions that aim to reduce risks of iatrogenic harm and improve health outcomes. Once tested, effective interventions need to be incorporated into local and international policy making in order to ensure that findings are effectively translated into practice.

## Conclusions

Family practice and pharmacy were identified as important contexts across all income categories. Particular areas identified as warranting further investigation included communication between health care professionals and with patients, teamwork within the health care team, laboratory and diagnostic imaging investigations, issues relating to data management, transitions between different care settings, and chart/patient record completeness. The WHO will be issuing a roadmap within the next 12 months to ensure that the momentum from this important initiative is maintained.

## Supporting Information

Text S1 **Sample data collection form.**
(DOCX)
