# Supplementary material for: Global Research Priorities to Better Understand the Burden of Iatrogenic Harm in Primary Care: An International Delphi Exercise
Source: PLoS Med. 2013 Nov 19;10(11):e1001554. doi: 10.1371/journal.pmed.1001554 (PMC3833831; doi:10.1371/journal.pmed.1001554)
Supplement: Text S1 — Sample data collection form. (DOCX) [file pmed.1001554.s001.docx]

**Supporting information: sample data collection form**

**Priority setting exercise: 3^rd^ round**

**Measuring the global burden of harm in primary care**

**INSTRUCTIONS HOW TO COMPLETE THE QUESTIONNAIRE**

We are aiming to reach agreement among a range of professional groups on the key components of unsafe health care in primary care settings. We need your help in identifying the issues that are of importance. In the following questionnaire, we have indicated how the panel members scored each item in Round 1. Please now score the items again, having had the chance to reflect on other panel members’ scores. The questionnaire is divided into three sections:

**SECTION 1:** Primary care contexts

**SECTION 2:** Areas that need to be focused on

**SECTION 3:** Conceptual/cross-cutting considerations

You are asked to score the importance of potential issues using the following grading:

**A 9-point scale with the anchors ‘‘not important’’ at 1 and ‘‘extremely important’’ at 9**

Please **circle the most appropriate score** in the **column next to each statement.**

Rest assured we will treat your responses in confidence.

**We would be grateful if you could complete the form as thoroughly as possible, but you are not obliged to complete all sections.**

If you have any comments, please use the space provided.

***[Definitions of patient safety, harm, primary care, and countries with different levels of income]***

**Topic: Measuring the frequency, associated disease burden and potential preventability of patient safety incidents in primary care**

**Scoring: A 9-point scale with the anchors ‘‘not important’’ at 1 and ‘‘extremely important’’ at 9**

| **SECTION 1**  **Statement: *‘‘The following contexts of primary care are important to develop a better understanding of the topic…’’*** | | | | | | | | | | | | | | | | |
| --- | --- | --- | --- | --- | --- | --- | --- | --- | --- | --- | --- | --- | --- | --- | --- | --- |
|  | **Please indicate the importance of the primary care context described as appropriate to answering the statement in the three income categories by circling a number from 1 to 9** | | | | | | | | | | | | | | | |
|  | **Low-income** | | | | **Middle-income** | | | | | | | | **High-income** | | | |
|  | **Median score Round 2** | **% agreement Round 2 for scores**  **7,8 & 9** | | **Your score Round 3** | **Median score Round 2** | | | **% agreement Round 2 for scores**  **7,8 & 9** | | | **Your score Round 3** | | **Median score Round 2** | | **% agreement Round 2 for scores**  **7,8 & 9** | **Your score Round 3** |
| Complementary therapies (e.g. acupuncture, herbal therapy) | 5 | 30.8 | | **1 2 3 4 5 6 7 8 9** | 4.5 | | | 25.0 | | | **1 2 3 4 5 6 7 8 9** | | 3 | | 12.0 | **1 2 3 4 5 6 7 8 9** |
| Community midwifery (maternity care) | 7 | 70.4 | | **1 2 3 4 5 6 7 8 9** | 7 | | | 75.9 | | | **1 2 3 4 5 6 7 8 9** | | 5 | | 20.0 | **1 2 3 4 5 6 7 8 9** |
| Pharmacy | 9 | 88.9 | | **1 2 3 4 5 6 7 8 9** | 9 | | | 96.4 | | | **1 2 3 4 5 6 7 8 9** | | 9 | | 87.5 | **1 2 3 4 5 6 7 8 9** |
| Community nursing | 7 | 69.2 | | **1 2 3 4 5 6 7 8 9** | 7 | | | 88.9 | | | **1 2 3 4 5 6 7 8 9** | | 6 | | 33.3 | **1 2 3 4 5 6 7 8 9** |
| Dentistry | 6 | 46.2 | | **1 2 3 4 5 6 7 8 9** | 6 | | | 46.2 | | | **1 2 3 4 5 6 7 8 9** | | 6.5 | | 50.0 | **1 2 3 4 5 6 7 8 9** |
| Family or general practice | 9 | 96.3 | | **1 2 3 4 5 6 7 8 9** | 9 | | | 100.0 | | | **1 2 3 4 5 6 7 8 9** | | 9 | | 96.2 | **1 2 3 4 5 6 7 8 9** |
| Home care/social work | 7 | 55.6 | | **1 2 3 4 5 6 7 8 9** | 7 | | | 64.3 | | | **1 2 3 4 5 6 7 8 9** | | 7 | | 69.2 | **1 2 3 4 5 6 7 8 9** |
| Occupational therapy | 5 | 19.2 | | **1 2 3 4 5 6 7 8 9** | 5 | | | 17.9 | | | **1 2 3 4 5 6 7 8 9** | | 5 | | 19.2 | **1 2 3 4 5 6 7 8 9** |
| Optometry | 5 | 25.0 | | **1 2 3 4 5 6 7 8 9** | 5 | | | 14.3 | | | **1 2 3 4 5 6 7 8 9** | | 5 | | 26.9 | **1 2 3 4 5 6 7 8 9** |
| Physiotherapy | 5 | 32.0 | | **1 2 3 4 5 6 7 8 9** | 5 | | | 14.3 | | | **1 2 3 4 5 6 7 8 9** | | 5.5 | | 34.6 | **1 2 3 4 5 6 7 8 9** |
| Podiatry | 4 | 20.0 | | **1 2 3 4 5 6 7 8 9** | 3 | | | 14.3 | | | **1 2 3 4 5 6 7 8 9** | | 4 | | 23.1 | **1 2 3 4 5 6 7 8 9** |
| Psychology | 6 | 44.4 | | **1 2 3 4 5 6 7 8 9** | 6 | | | 28.6 | | | **1 2 3 4 5 6 7 8 9** | | 6 | | 46.2 | **1 2 3 4 5 6 7 8 9** |
| Speech therapy | 4 | 15.4 | | **1 2 3 4 5 6 7 8 9** | 3 | | | 7.1 | | | **1 2 3 4 5 6 7 8 9** | | 3 | | 15.4 | **1 2 3 4 5 6 7 8 9** |
| Nutritionists | 6 | 29.6 | | **1 2 3 4 5 6 7 8 9** | 6 | | | 28.6 | | | **1 2 3 4 5 6 7 8 9** | | 5 | | 16.0 | **1 2 3 4 5 6 7 8 9** |
| Health inspectors | 7 | 60.0 | | **1 2 3 4 5 6 7 8 9** | 7 | | | 75.0 | | | **1 2 3 4 5 6 7 8 9** | | 5 | | 26.9 | **1 2 3 4 5 6 7 8 9** |
| Care homes | 6 | 42.3 | | **1 2 3 4 5 6 7 8 9** | 6 | | | 40.7 | | | **1 2 3 4 5 6 7 8 9** | | 8 | | 84.0 | **1 2 3 4 5 6 7 8 9** |
| Community health volunteers | 5 | 40.0 | | **1 2 3 4 5 6 7 8 9** | 6 | | | 42.9 | | | **1 2 3 4 5 6 7 8 9** | | 3 | | 7.7 | **1 2 3 4 5 6 7 8 9** |
| Traditional medicine (e.g. witchdoctors) | 5.5 | 38.5 | | **1 2 3 4 5 6 7 8 9** | 5 | | | 11.1 | | | **1 2 3 4 5 6 7 8 9** | | 1 | | 0.0 | **1 2 3 4 5 6 7 8 9** |
| Schools | 5 | 41.7 | | **1 2 3 4 5 6 7 8 9** | 5 | | | 25.0 | | | **1 2 3 4 5 6 7 8 9** | | 3 | | 19.2 | **1 2 3 4 5 6 7 8 9** |
| **SECTION 2**  **Statement: *“The following causes of patient safety incidents and their associated harm need to be studied further…”*** | | | | | | | | | | | | | | | | |
|  | | **Please indicate the importance of the cause described in answering the statement in the three income categories by circling a number from 1 to 9** | | | | | | | | | | | | | | |
|  | | **Low-income** | | | | | **Middle-income** | | | | | **High-income** | | | | |
|  | | **Median score Round 2** | **% agreement Round 2 for scores**  **7,8 & 9** | | **Your score Round 3** | | **Median score Round 2** | | **% agreement Round 2 for scores**  **7,8 & 9** | **Your score Round 3** | | **Median score Round 2** | | | **% agreement Round 2 for scores**  **7,8 & 9** | **Your score Round 3** |
| **Office administration (overall)** | | 6 | 42.3 | | **1 2 3 4 5 6 7 8 9** | | 7 | | 80.8 | **1 2 3 4 5 6 7 8 9** | | 7 | | | 76.0 | **1 2 3 4 5 6 7 8 9** |
| Filing | | 6.5 | 50.0 | | **1 2 3 4 5 6 7 8 9** | | 7 | | 59.3 | **1 2 3 4 5 6 7 8 9** | | 7 | | | 68.0 | **1 2 3 4 5 6 7 8 9** |
| Chart/patient record completeness | | 8 | 65.4 | | **1 2 3 4 5 6 7 8 9** | | 8 | | 85.2 | **1 2 3 4 5 6 7 8 9** | | 8 | | | 84.0 | **1 2 3 4 5 6 7 8 9** |
| Patient flow | | 6.5 | 50.0 | | **1 2 3 4 5 6 7 8 9** | | 7 | | 73.1 | **1 2 3 4 5 6 7 8 9** | | 7 | | | 66.7 | **1 2 3 4 5 6 7 8 9** |
| Message handling | | 7 | 57.7 | | **1 2 3 4 5 6 7 8 9** | | 7 | | 70.4 | **1 2 3 4 5 6 7 8 9** | | 7 | | | 72.0 | **1 2 3 4 5 6 7 8 9** |
| Appointments | | 5 | 30.8 | | **1 2 3 4 5 6 7 8 9** | | 5 | | 33.3 | **1 2 3 4 5 6 7 8 9** | | 6 | | | 48.0 | **1 2 3 4 5 6 7 8 9** |
| **Communication (overall)** | | 9 | 88.5 | | **1 2 3 4 5 6 7 8 9** | | 9 | | 96.4 | **1 2 3 4 5 6 7 8 9** | | 9 | | | 100.0 | **1 2 3 4 5 6 7 8 9** |
| Between healthcare professionals in the same team | | 9 | 81.5 | | **1 2 3 4 5 6 7 8 9** | | 9 | | 92.3 | **1 2 3 4 5 6 7 8 9** | | 9 | | | 91.3 | **1 2 3 4 5 6 7 8 9** |
| Between healthcare professionals from different teams/settings (e.g. referrals) | | 9 | 81.5 | | **1 2 3 4 5 6 7 8 9** | | 9 | | 92.9 | **1 2 3 4 5 6 7 8 9** | | 9 | | | 100.0 | **1 2 3 4 5 6 7 8 9** |
| Between healthcare professionals and patients | | 9 | 88.9 | | **1 2 3 4 5 6 7 8 9** | | 9 | | 100.0 | **1 2 3 4 5 6 7 8 9** | | 9 | | | 95.8 | **1 2 3 4 5 6 7 8 9** |
| Teamwork | | 8 | 77.8 | | **1 2 3 4 5 6 7 8 9** | | 9 | | 100.0 | **1 2 3 4 5 6 7 8 9** | | 9 | | | 92.3 | **1 2 3 4 5 6 7 8 9** |
| **Investigations (overall)** | | 8 | 75.0 | | **1 2 3 4 5 6 7 8 9** | | 8 | | 96.3 | **1 2 3 4 5 6 7 8 9** | | 8 | | | 96.2 | **1 2 3 4 5 6 7 8 9** |
| Laboratory | | 8 | 70.4 | | **1 2 3 4 5 6 7 8 9** | | 8 | | 85.2 | **1 2 3 4 5 6 7 8 9** | | 8 | | | 96.2 | **1 2 3 4 5 6 7 8 9** |
| Diagnostic imaging | | 7 | 63.0 | | **1 2 3 4 5 6 7 8 9** | | 7 | | 74.1 | **1 2 3 4 5 6 7 8 9** | | 8 | | | 88.5 | **1 2 3 4 5 6 7 8 9** |
| Other investigations | | 6 | 47.8 | | **1 2 3 4 5 6 7 8 9** | | 7 | | 70.8 | **1 2 3 4 5 6 7 8 9** | | 8 | | | 77.3 | **1 2 3 4 5 6 7 8 9** |
| **Medications (overall)** | | 9 | 100.0 | | **1 2 3 4 5 6 7 8 9** | | 9 | | 96.3 | **1 2 3 4 5 6 7 8 9** | | 9 | | | 100.0 | **1 2 3 4 5 6 7 8 9** |
| Counterfeit drugs | | 9 | 96.3 | | **1 2 3 4 5 6 7 8 9** | | 8 | | 81.5 | **1 2 3 4 5 6 7 8 9** | | 5 | | | 26.9 | **1 2 3 4 5 6 7 8 9** |
| **Knowledge/clinical errors (overall)** | | 9 | 92.6 | | **1 2 3 4 5 6 7 8 9** | | 9 | | 96.2 | **1 2 3 4 5 6 7 8 9** | | 8 | | | 76.0 | **1 2 3 4 5 6 7 8 9** |
| Execution of a clinical task (errors when performing clinical tasks due to lack of knowledge and/or skills) | | 9 | 96.3 | | **1 2 3 4 5 6 7 8 9** | | 8 | | 96.2 | **1 2 3 4 5 6 7 8 9** | | 8 | | | 76.9 | **1 2 3 4 5 6 7 8 9** |
| Wrong or missed diagnoses | | 9 | 92.6 | | **1 2 3 4 5 6 7 8 9** | | 9 | | 96.3 | **1 2 3 4 5 6 7 8 9** | | 9 | | | 100.0 | **1 2 3 4 5 6 7 8 9** |
| Wrong treatment decision | | 9 | 92.6 | | **1 2 3 4 5 6 7 8 9** | | 9 | | 92.9 | **1 2 3 4 5 6 7 8 9** | | 8 | | | 88.5 | **1 2 3 4 5 6 7 8 9** |
| **Payment (errors in process of payment)** | | 5 | 26.9 | | **1 2 3 4 5 6 7 8 9** | | 5 | | 13.0 | **1 2 3 4 5 6 7 8 9** | | 5 | | | 12.0 | **1 2 3 4 5 6 7 8 9** |
| **Information technology and tools (e.g. checklists)** | | 7 | 55.6 | | **1 2 3 4 5 6 7 8 9** | | 8 | | 85.2 | **1 2 3 4 5 6 7 8 9** | | 8 | | | 88.5 | **1 2 3 4 5 6 7 8 9** |
| **Transitions between different levels of care** | | 9 | 74.1 | | **1 2 3 4 5 6 7 8 9** | | 9 | | 96.4 | **1 2 3 4 5 6 7 8 9** | | 9 | | | 92.3 | **1 2 3 4 5 6 7 8 9** |
| **Higher-level systems management (e.g. human resources)** | | 8 | 70.4 | | **1 2 3 4 5 6 7 8 9** | | 8 | | 84.6 | **1 2 3 4 5 6 7 8 9** | | 8 | | | 88.0 | **1 2 3 4 5 6 7 8 9** |
| **Data management** | | 8 | 70.4 | | **1 2 3 4 5 6 7 8 9** | | 8 | | 92.9 | **1 2 3 4 5 6 7 8 9** | | 8 | | | 96.2 | **1 2 3 4 5 6 7 8 9** |
| **SECTION 3**  **Statement: *“The following cross-cutting topics are important to focus on …”*** | | | | | | | | | | | | | | | | |
|  | | | | | | **Please indicate the importance of the cross-cutting topic described by circling a number from 1 to 9** | | | | | | | | | | |
|  | | | | | | **Median score Round 2** | | | | **% agreement Round 2 for scores**  **7,8 & 9** | | | | **Your score Round 3** | | |
| Greater clarity on definitions | | | | | | 8 | | | | 84.8 | | | | **1 2 3 4 5 6 7 8 9** | | |
| Improved typologies / taxonomies | | | | | | 8 | | | | 81.8 | | | | **1 2 3 4 5 6 7 8 9** | | |
| Statement on quality of reporting of studies (e.g. CONSORT) | | | | | | 7 | | | | 55.2 | | | | **1 2 3 4 5 6 7 8 9** | | |
| System to share errors | | | | | | 8 | | | | 90.3 | | | | **1 2 3 4 5 6 7 8 9** | | |
| Advocacy | | | | | | 8 | | | | 81.8 | | | | **1 2 3 4 5 6 7 8 9** | | |
| Policy making | | | | | | 8 | | | | 81.8 | | | | **1 2 3 4 5 6 7 8 9** | | |
| Education and training | | | | | | 9 | | | | 97.0 | | | | **1 2 3 4 5 6 7 8 9** | | |
| Regulations | | | | | | 8 | | | | 84.4 | | | | **1 2 3 4 5 6 7 8 9** | | |
| Data collection methods | | | | | | 8 | | | | 87.5 | | | | **1 2 3 4 5 6 7 8 9** | | |
| Incentives | | | | | | 7 | | | | 60.6 | | | | **1 2 3 4 5 6 7 8 9** | | |

Please use this space for any additional comments

**We are very grateful** **for your help with this priority setting exercise.**
